# Delayed Diagnosis of an Occult Femoral Neck Fracture Undetected by MRI and Dual-Energy CT: A Case Report

**DOI:** 10.7759/cureus.110951

**Published:** 2026-06-16

**Authors:** Akihiro Odagaki, Takahiro Oda, Kenjiro Iwasa, Kazuki Kodato, Takahiro Niikura

**Affiliations:** 1 Orthopaedic Surgery, Hyogo Prefectural Nishinomiya Hospital, Nishinomiya, JPN

**Keywords:** bone marrow alteration, bone marrow edema, delayed diagnosis, dual-energy computed tomography (dect), false-negative imaging, femoral neck fracture, hip pain, magnetic resonance imaging (mri), myelodysplastic syndrome, occult fracture

## Abstract

Although MRI and dual-energy CT (DECT) are considered highly sensitive tools for detecting bone marrow edema and subtle fractures, occult femoral neck fractures may still be missed. Here, we report a rare case in which an occult femoral neck fracture was not detected on MRI or DECT during the initial evaluation. A 64-year-old woman with myelodysplastic syndrome (MDS) and residual left hemiparesis presented with persistent left hip pain after a fall. Initial radiography, CT, MRI, and DECT revealed no evidence of fractures or bone marrow edema. Despite conservative management, the patient’s symptoms persisted, and repeat imaging one month later revealed a non-displaced subcapital femoral neck fracture. The patient underwent internal fixation using the Femoral Neck System (DePuy Synthes, Johnson & Johnson, Raynham, MA, USA) combined with a cannulated cancellous screw and had an uneventful postoperative course. The absence of typical MRI findings was likely related to diffuse bone marrow alterations associated with MDS, which may have obscured fracture-related edema. DECT can also fail to detect early or subtle trabecular injuries, particularly when the imaging parameters are suboptimal. Occult femoral neck fractures may remain undetectable with MRI and DECT, particularly in patients with hematologic disorders affecting the bone marrow composition. Persistent hip pain, despite negative imaging findings, should prompt repeat or parameter-optimized imaging.

## Introduction

Femoral neck fractures are common injuries in elderly patients and are associated with significant morbidity and mortality if not promptly diagnosed and treated [1,2]. Early and accurate detection is crucial for preventing secondary displacement, avascular necrosis, and prolonged functional impairment. However, some fractures are radiographically occult at the initial presentation, leading to diagnostic delays and unfavorable outcomes.

MRI is widely considered the gold standard for detecting occult femoral neck fractures because of its high sensitivity for bone marrow edema and subtle cortical disruptions [3,4]. Recently, dual-energy CT (DECT) was reported to visualize bone marrow changes and microtrabecular injuries with considerable diagnostic accuracy [3,5,6]. Nevertheless, false-negative findings may occur in the early phase after injury or in patients with underlying conditions that alter the bone marrow signal characteristics, such as hematological disorders [7,8].

Here, we report a case of an occult femoral neck fracture that remained undetected on MRI and DECT in a patient with altered bone marrow composition and discuss the potential mechanisms of false-negative findings and their implications for clinical management.

## Case presentation

A 64-year-old woman with a history of myelodysplastic syndrome (MDS) and residual left hemiparesis secondary to a cerebral infarction presented with left hip pain after falling during a hospital visit. The patient received regular follow-up at the hematology outpatient clinic of our institution. At presentation, the patient had difficulty ambulating due to weight-bearing pain in the left lower limb and required a wheelchair for mobility.

Physical examination revealed no swelling or ecchymosis in the left thigh. However, localized tenderness was observed over the lateral aspect of the left hip. Active straight leg raising of the left lower limb was impossible because of pain.

Plain radiographs of the pelvis and hip revealed no evidence of fractures or dislocations (Figure 1). Noncontrast CT performed on the same day showed no cortical disruption or fracture lines (Figure 2). MRI revealed diffusely low signal intensity throughout the proximal femur on T1-weighted images, except for the weight-bearing area, without a definite fracture line at the time of the initial interpretation (Figure 3A). Fat-suppressed T2-weighted images showed no obvious high-signal change suggestive of bone marrow edema, and no fracture line was prospectively recognized, although a subtle linear structure can be appreciated on retrospective review (Figure 3B). Concurrent DECT revealed no increase in bone marrow attenuation or cortical discontinuities (Figure 4).

**Figure 1:**
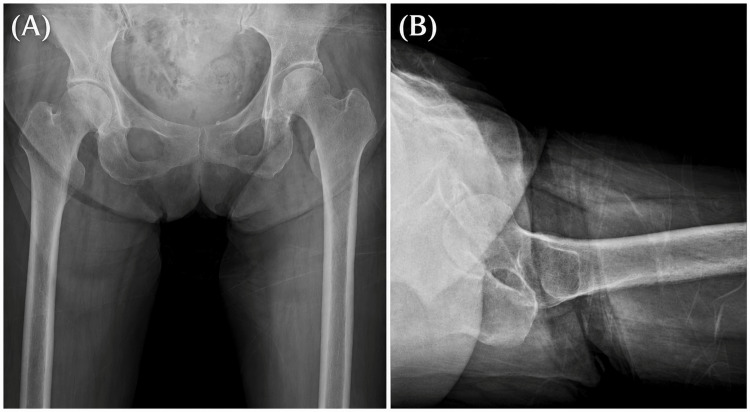
Initial plain radiographs of the hip. (A) Anteroposterior view; (B) Axial (cross-table lateral) view. No obvious fracture is observable in the pelvis or left hip.

**Figure 2:**
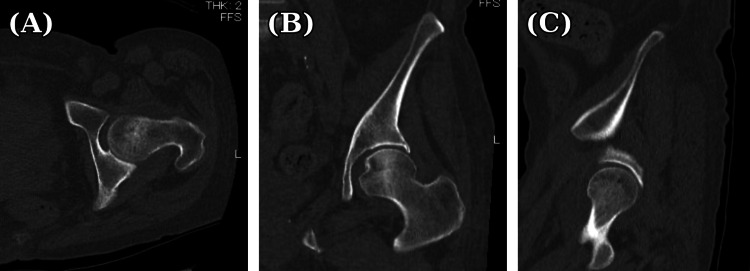
Initial single-energy CT images of the left hip. (A) Axial view; (B) Coronal view; (C) Sagittal view. No interruption of cortical bone continuity or fracture line is observable.

**Figure 3:**
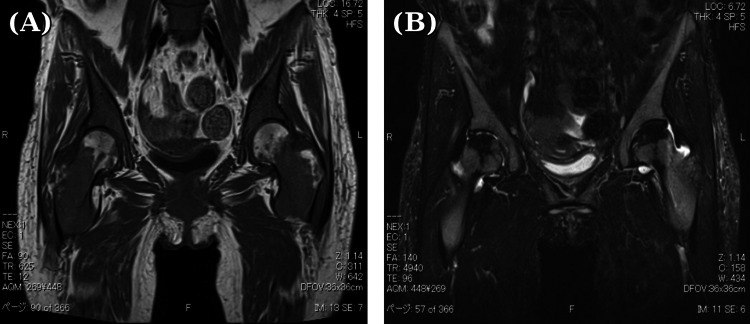
MRI of the left hip at the initial evaluation. (A) T1-weighted image showing diffusely low signal intensity throughout the proximal femur, except for the weight-bearing area, without a prospectively identifiable fracture line. (B) Fat-suppressed T2-weighted image showing no obvious high-signal change suggestive of bone marrow edema; a subtle vertical linear structure can be retrospectively appreciated at the subcapital region, but was not recognized as a definite fracture line at the initial interpretation.

**Figure 4:**
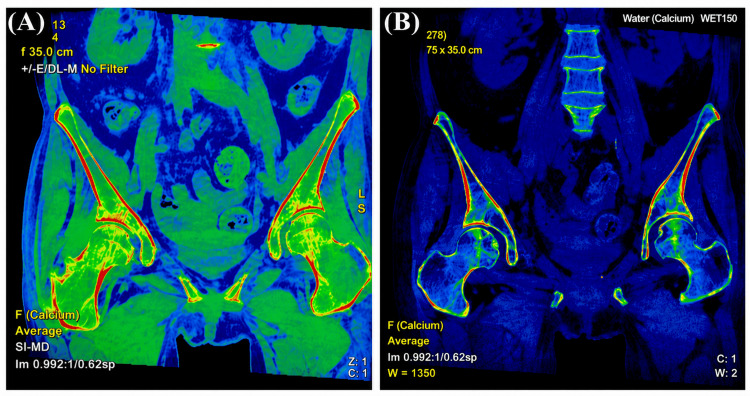
Dual-energy CT (coronal) images of the hip obtained at the initial evaluation. (A) Water–calcium material density map; (B) Water–hydroxyapatite material density map. No changes suggestive of fracture are observable in either image.

Despite conservative treatment and observation, the patient continued to experience persistent hip pain, particularly during weight-bearing activities, and their functional status did not improve. Accordingly, weekly outpatient follow-up was continued. One month after the injury, repeat plain radiography and CT revealed a distinct fracture line in the subcapital region of the femoral neck that had not been apparent initially (Figures 5, 6). 

**Figure 5:**
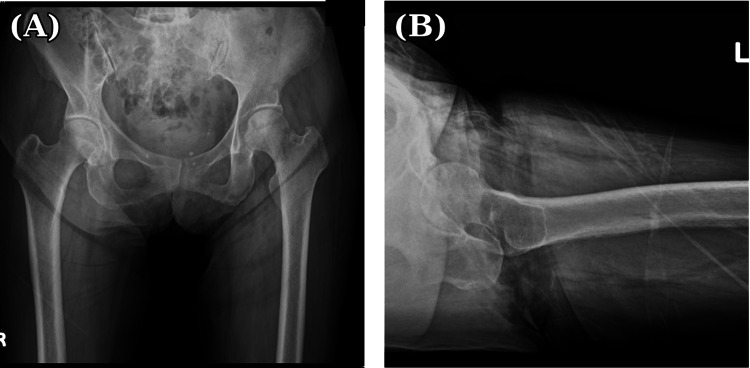
Plain radiographs obtained one month after the injury. (A) Anteroposterior view; (B) Axial view. The images show a distinct subcapital femoral neck fracture line that was not visible initially.

**Figure 6:**
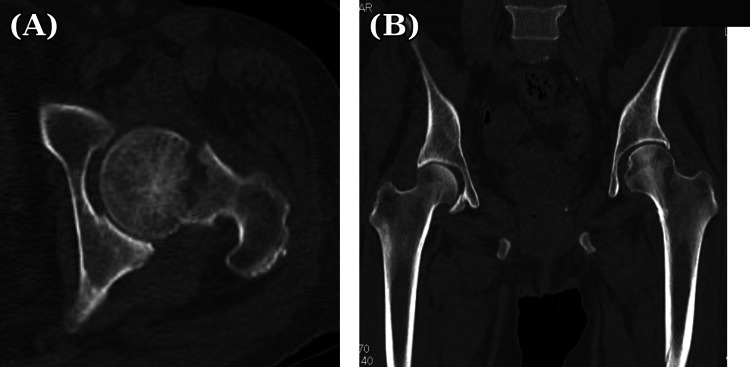
CT images of the left hip obtained one month after the injury. (A) Axial view; (B) Coronal view. A clear fracture line is observed at the subcapital region of the femoral neck.

A nondisplaced femoral neck fracture was diagnosed, and internal fixation was performed the same day using the Femoral Neck System (DePuy Synthes, Johnson & Johnson, Raynham, MA, USA) combined with a 6.5-mm × 75-mm cannulated cancellous screw (Meira Corporation, Nagoya, Japan) (Figure 7). 

**Figure 7:**
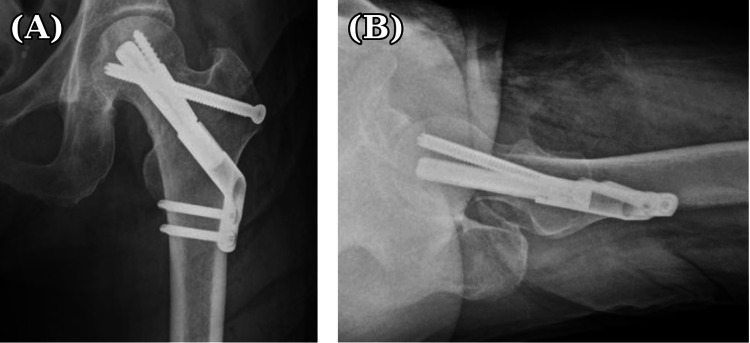
Postoperative plain radiographs. (A) Anteroposterior view; (B) Axial view. The images confirm that the internal fixation with the Femoral Neck System (DePuy Synthes, Johnson & Johnson, Raynham, MA, USA) and a cannulated cancellous screw (Meira Corporation, Nagoya, Japan) are in appropriate alignment, with no displacement or other complications.

The postoperative course was uneventful. Three months postoperatively, the patient was pain-free and had returned to their pre-injury level of mobility and activities of daily living without complications.

## Discussion

Occult femoral neck fractures can be difficult to diagnose at the initial presentation, even when advanced imaging modalities are used. MRI is widely recognized as the most sensitive imaging modality for the detection of occult fractures, with previous studies reporting nearly 100% sensitivity and specificity [3,4]. Typical MRI findings include a low-signal fracture line on T1-weighted images and high-signal bone marrow edema on T2-weighted or fat-suppressed images. In the present case, however, these characteristic findings were absent despite the MRI being performed shortly after the injury.

This case suggests that underlying hematologic disorders, such as MDS, may complicate the interpretation of MRI findings in patients with suspected occult fractures. In patients with MDS, the normal fatty marrow may be replaced by hypercellular hematopoietic tissue, resulting in diffusely low signal intensity on T1-weighted images and altered signal characteristics on T2-weighted images [7,8]. Consequently, localized marrow signal changes related to a fracture may be difficult to distinguish from diffuse marrow signal abnormalities, potentially leading to false-negative or indeterminate interpretations. In the present case, such diffuse marrow signal alteration may have contributed to the difficulty in prospectively identifying a subtle fracture-related signal change on the initial MRI.

Quantitative MRI assessment may help distinguish fracture-related marrow edema from hematological marrow alterations. Signal intensity drop ratio analysis using opposed-phase imaging is useful for estimating residual fat components within the bone marrow. A signal intensity drop rate of less than 20% is generally associated with hematologic marrow conversion, whereas a rate greater than 20% suggests edema-related changes, such as those observed in occult fractures. [8] In the present case, the diffusely low and homogeneous signal pattern without focal edema was more consistent with MDS-related marrow alterations than with fracture-associated changes.

Diffusion-weighted imaging and apparent diffusion coefficient (ADC) mapping may provide complementary diagnostic information when conventional MRI is inconclusive. The increased marrow cellularity caused by MDS typically restricts diffusion and results in low ADC values, whereas fracture-related bone marrow edema is characterized by increased extracellular water content and elevated ADC values. The absence of diffusion-weighted imaging and ADC evaluations in the initial MRI protocol may have contributed to the delayed diagnosis in this case [9].

This case also highlights the limitations of DECT in the early detection of occult femoral neck fractures. Although DECT has been reported to visualize bone marrow edema and microtrabecular injuries with high diagnostic accuracy, its sensitivity may be reduced in the early phase after injury or when post-processing parameters are suboptimal [5,6]. In addition, marrow changes related to hematological disorders may produce attenuation patterns that resemble pathological infiltration, potentially obscuring subtle fracture-related findings.

Overall, this case underscores that MRI and DECT, despite their reportedly high sensitivity, may yield false-negative results in patients with altered bone marrow composition. When the clinical suspicion remains high despite negative imaging findings, repeat imaging or parameter-optimized MRI and DECT protocols should be considered to avoid delayed diagnosis and subsequent complications [10,11].

## Conclusions

This case illustrates that occult femoral neck fractures may remain undetectable even when evaluated using MRI and DECT. The difficulty in diagnosing this patient was likely related to diffuse bone marrow alterations caused by MDS, which can mask the typical MRI features of bone marrow edema. Although MRI and DECT are regarded as highly sensitive modalities, their diagnostic performance may be limited in patients with hematologic disorders that alter the marrow composition. Optimization of MRI parameters, including the assessment of signal intensity ratios and the use of diffusion-weighted imaging, together with appropriate adjustment of DECT reconstruction conditions, may improve early fracture detection. Clinicians should remain vigilant and consider repeat or parameter-optimized imaging when hip pain persists despite negative initial findings.

## References

[1] Emmerson BR, Varacallo MA, Inman D (2026). Hip Fracture Overview. https://www.ncbi.nlm.nih.gov/books/NBK557514/.

[2] Parker MJ, Gurusamy K (2006). Internal fixation versus arthroplasty for intracapsular proximal femoral fractures in adults. Cochrane Database Syst Rev.

[3] Cabarrus MC, Ambekar A, Lu Y, Link TM (2008). MRI and CT of insufficiency fractures of the pelvis and the proximal femur. AJR Am J Roentgenol.

[4] Pandey R, McNally E, Ali A, Bulstrode C (1998). The role of MRI in the diagnosis of occult hip fractures. Injury.

[5] Wang CK, Tsai JM, Chuang MT, Wang MT, Huang KY, Lin RM (2013). Bone marrow edema in vertebral compression fractures: detection with dual-energy CT. Radiology.

[6] Guggenberger R, Gnannt R, Hodler J (2012). Diagnostic performance of dual-energy CT for the detection of traumatic bone marrow lesions in the ankle: comparison with MR imaging. Radiology.

[7] Vande Berg BC, Malghem J, Lecouvet FE, Maldague B (1998). Magnetic resonance imaging of normal bone marrow. Eur Radiol.

[8] Mourad C, Cosentino A, Nicod Lalonde M, Omoumi P (2023). Advances in bone marrow imaging: strengths and limitations from a clinical perspective. Semin Musculoskelet Radiol.

[9] Frihagen F, Nordsletten L, Tariq R, Madsen JE (2005). MRI diagnosis of occult hip fractures. Acta Orthop.

[10] Dietrich O, Biffar A, Reiser MF, Baur-Melnyk A (2009). Diffusion-weighted imaging of bone marrow. Semin Musculoskelet Radiol.

[11] Blake SP, Connors AM (2004). Sacral insufficiency fracture. Br J Radiol.

